# Revisiting the past in light of current knowledge on γδ T cells in leishmaniasis

**DOI:** 10.3389/fimmu.2026.1684708

**Published:** 2026-03-20

**Authors:** Júlio Souza dos-Santos, Herbert Leonel de Matos Guedes

**Affiliations:** 1 Immunobiotechnology Laboratory, Institute of Microbiology Paulo de Góes, Federal University of Rio de Janeiro, Rio de Janeiro, RJ, Brazil; 2 Clinical Immunology Laboratory, Oswaldo Cruz Institute, Oswaldo Cruz Foundation (Fiocruz), Rio de Janeiro, RJ, Brazil

**Keywords:** human leishmaniasis, experimental leishmaniasis, γδ T cells, treatment outcomes, leishmaniasis duration

## Abstract

Leishmaniasis, a group of neglected diseases caused by *Leishmania* parasites, presents complex immune responses shaped by parasite strain, disease type, treatment regimens, and experimental models. Among the immune players, γδ T cells have gained significant attention due to their dual role in producing pro-inflammatory cytokines like Interleukin (IL)-17 and Interferon (IFN)-γ, alongside their cytotoxic functions. These cells play pivotal roles in various diseases, including cancer and malaria, and their impact on leishmaniasis is increasingly recognized. Since their identification in patient lesions in 1989, γδ T cells have been shown to influence disease progression in leishmaniasis. However, their role remains nuanced, with a delicate balance between IL-17, IL-10, and IFN-γ production, each cytokine modulating the expression of others. In this review, we explore how γδ T cells shape the course of leishmaniasis in humans, affecting both disease outcomes and treatment responses. We also highlight significant differences between species and experimental models, which critically impact infection dynamics. Furthermore, we emphasize probable ligands present on *Leishmania* parasites that may activate γδ T cells, providing insights into potential mechanisms of immune recognition and response. Additionally, we examine the sublocalization of γδ T cells across various tissues, providing a detailed view of their distribution in the context of leishmaniasis. These insights raise crucial considerations for advancing disease control strategies and the development of innovative therapeutic approaches.

## Introduction

γδ T cells are lymphocytes mainly developed in the thymus and exhibit an intrinsic property of appearing in sequential waves during development, which shapes the formation of distinct subsets that vary in their cytokine profiles, including IL-17 and IFN-γ, as well as their distribution across different tissues. This characteristic has been observed in both mice and humans (1–4). In humans, γδ T cell subsets are typically categorized by the TCRδ chain, which defines two main subsets: Vδ1 and Vδ2, along with less common populations expressing Vδ3. Vδ1 cells are predominantly found in tissues, whereas Vδ2 cells, which can express Vγ9, are mainly present in the blood (5). In mice, γδ T cells are classified based on the type of Vγ family they use (Vγ1, Vγ2, Vγ4, Vγ5, Vγ6, and Vγ7), according to the nomenclature of Tonegawa; and reside in a variety of organs (6).

As development progresses, αβ T cells emerge later from the thymus, giving rise to the conventional CD4^+^ and CD8^+^ lineages that dominate adaptive immune responses (7). Thus, the T cell receptor (TCR) complex consists of the variable αβ or γδ chains associated with the invariant CD3 subunits (CD3ϵ, CD3γ, CD3δ, and CD3ζ) (8). In humans, both αβ and γδ TCRs share the same composition (TCRαβCD3ϵ_2_γδζ_2_ or TCRγδCD3ϵ_2_γδζ_2_). In contrast, murine γδ TCRs lack CD3δ and incorporate two CD3γ subunits (TCRγδCD3ϵ_2_γ_2_ζ_2_) (8). Notably, γδ T cells can be divided into two populations based on CD3 expression levels, CD3^high^ and CD3^low^, and this distinction directly influences their capacity to produce IL-17 under different immunological contexts (9–16). Besides, the structural difference explains why human CD3δ deficiency blocks the development of both αβ and γδ T cells, while in mice it affects only αβ T cells, since murine γδ TCRs do not require CD3δ for surface expression (8). However, CD3δ may still aid γδ TCR assembly in the endoplasmic reticulum. Additionally, both αβ and γδ TCRs can exchange CD3ζ for FcR-γ, reducing signaling strength due to the lower ITAM number in FcR-γ (8). Thus, the distinct CD3 composition between αβ and γδ TCRs, especially the absence of CD3δ in murine γδ T cells, reveals species-specific adaptations that impact T cell development and signaling efficiency.

Although the human Vγ and Vδ repertoires are considerably smaller than those of TCRα and TCRβ (17), the TCR-δ chain frequently undergoes extensive N-region nucleotide additions across its D segments, which can be translated in any reading frame. This junctional variability generates hyperdiversity concentrated within the CDR3 loops, which are crucial for determining antigen specificity (18, 19). Consequently, the theoretical number of distinct γδ TCRs that could be generated is potentially comparable to that of αβ TCRs (18, 19). In contrast to the α and β chains, whose CDR3 length is constrained by the requirement for precise interactions with peptide–MHC complexes, the δ-chain CDR3 is typically longer and exhibits greater variability than its γ-chain counterpart (20–23). Beyond this, the γδ TCR displays a unique feature in which the TCRγ chain itself plays a major role in shaping γδ TCR reactivity. This stems from the interaction between the Vγ chain and BTNL molecules, which involves amino acids located within and around the germline-encoded CDR2 region and the hypervariable region 4 (HV4)—an additional hypervariable segment shared by TCR and immunoglobulin gene families (21–23). Thus, despite possessing a smaller overall repertoire compared to αβ TCRs, the γδ TCR can recognize an equally broad range of molecular targets through distinct modes of interaction. Specifically, γδ TCRs engage ligands via two principal mechanisms: a non-clonotypic mode mediated by BTNL/BTN molecules that regulates specific γδ T cell subsets, and a CDR3-dependent, antibody-like mode that confers adaptive-like precision to γδ T cell responses (21–23).

Unlike αβ T cells, which acquire their effector functions only after peripheral activation, γδ T cells emerge from the thymus already pre-programmed for effector activity, with a predetermined capacity to produce cytokines (7). Moreover, one of the main distinctions between γδ and αβ T cells is that most γδ T cells do not rely on classical MHC (Major Histocompatibility Complex)-mediated antigen presentation (24, 25). Instead, they can recognize a broad range of antigens of different natures presented by non-classical MHC molecules, Cluster of Differentiation 1 (CD1), and MHC-related protein (MR1), enabling them to act rapidly and flexibly in both innate and adaptive immune responses (7). This underscores the need for identifying the ligands that activate γδ T cells via the γδ TCR. γδ T cells can recognize lipid metabolites induced by stress through CD1d receptor presentation (26, 27), as well as through the sensing ligands BTN2A1 (Butyrophilin Subfamily 2 Member A1) (23, 28) and BTN3A1 (29–31), but an open question remains regarding the ability of γδ T cells to recognize pathogen-specific antigens. However, it has been reported that LPG (lipophosphoglycan) from different *Leishmania* species can activate γδ T cells (12, 32). Specifically in *L. mexicana*, stimulation with purified LPG induced TLR2 activation in γδ T cells. Upon inoculation of *L. mexicana* parasites or purified LPG into the ear dermis, γδ T cells through TLR2 binding LPG were observed, confirming a direct interaction during infection (32). Interestingly, a subset of LPG^+^ but TLR2^−^ γδ T cells indicated that additional, yet unidentified receptors may also recognize LPG (32). Together, these findings suggest that LPG acts as a key molecule in the activation and modulation of γδ T cells during *L. mexicana* infection, engaging both TLR2-dependent and alternative recognition pathways.

Leishmaniasis comprises a group of immunologically complex diseases caused by various *Leishmania* parasite subspecies. Infection with these protozoa results in cutaneous and/or mucocutaneous diseases, collectively referred to as Cutaneous Leishmaniasis (CL), as well as visceral diseases, known as Visceral Leishmaniasis (VL), found in both the Old and New Worlds. Affecting primarily impoverished populations, leishmaniasis is considered a neglected disease, impacting over 90 countries across Asia, Africa, the Middle East, and Central and South America (33). The immunology of leishmaniasis has been extensively studied in murine animal models, which have contributed to the elucidation of the T helper (Th)1/Th2 paradigm that influences T cell responses during infection with *Leishmania* species. This is most evident in *L. major* infection, characterized by a Th1 response, leading to IFN-γ production in resistant C57BL/6 mice, and a Th2 response with IL-4 generation in susceptible BALB/c mice, resulting in a state of susceptibility (34). However, it is known that other *Leishmania* species involve a combination of various immune elements. A mixed Th1/Th2 response, characterized by the expression of IFN-γ, Tumor Necrosis Factor (TNF)-α, IL-12 and IL-4, is observed in active lesions; however, a robust Th1 immune profile, driven by IFN-γ induction, is primarily responsible for clinical cure (35).

It is important to note that in lesions from patients with diffuse cutaneous leishmaniasis caused by *L. amazonensis* for over three decades, no induction of cytokine transcripts related to the Th2 profile is observed (36). This finding raises the discussion that an immune environment skewed toward a Th2 response is not solely required for the persistence of refractory disease in these patients. Therefore, it is evident that further investigation is needed to identify other cell types that contribute to the pathogenesis of this disease. Several studies have described the presence of γδ T cells in varying quantities in both cutaneous and visceral leishmaniasis, regardless of the clinical characteristics of the diseases, including their duration (37–41). In experimental leishmaniasis, the data are highly conflicting, depending on the parasite strain and animal model used (12, 42–44). In this review, we aim to thoroughly investigate the role of γδ T cells in leishmaniasis, offering a new perspective on how these cells may directly impact disease outcomes.

## γδ T cells in human leishmaniasis

### Human cutaneous leishmaniasis

The presence of γδ T cells has been observed in lesions of patients infected with *L. braziliensis*, associated with cutaneous, mucocutaneous and diffuse leishmaniasis (45, 46). One of the first work (45) identified that lesions of *L. braziliensis*-infected patients, characterized by granulomatous skin reactions, have around 20% of the CD3^+^ cells in these lesions stained positive for anti-TCRδ1, however, this frequency could be maintained at an average of 1-5% of T cell present in the lesion (46, 47). The presence of cells TCRδ1^+^ was limited to less than 5% in the peripheral blood of the same patients (45), along with Montenegro skin reactions and in the lesions of those with mucocutaneous leishmaniasis. Indeed, γδ T cells appear to be more abundant in CL than in Diffuse Cutaneous Leishmaniasis (DCL) and Mucocutaneous Leishmaniasis (MC) (46). This distribution suggests that γδ T cells preferentially accumulate at the site of infection, where they may participate in early immune activation and tissue organization. The higher frequency observed in CL compared to DCL and MC indicates that the presence of γδ T cells may correlate with better containment of the parasite and a more effective local immune response.

The suppression of αβ T cell responses mediated by infection with *Leishmania* species may be one reason for the expansion of γδ T cells (48). In fact, γδ T cells undergo homeostatic expansion in T cell-deficient environment (49). The immunosuppression has been associated with cases of hepatosplenic γδ T-cell lymphoma in humans (50) and extranodal γδ T-cell lymphoma in dogs (51). In both *L. braziliensis* and *L. amazonensis* infections, there are higher levels of T cells expressing IL-10 and Transforming Growth Factor (TGF)-β compared to IFN-γ and TNF-α in Peripheral Blood Mononuclear Cells (PBMCs) from patient lesions (52, 53). In patients with visceral leishmaniasis, γδ T cells produce a mixture of Th1/Th2 cytokines such as IL-2, IL-4, TNF- α, IFN-γ, as well as IL-10 (54). The αβ Double Negative (DN - CD4^-^CD8^-^) T cells from PBMCs of CL patients exhibit an intensified inflammatory cytokine profile, producing IFN-γ and TNF-α after stimulation with soluble leishmanial antigen (SLA) from *L. braziliensis*, while the γδ DN T cells display a regulatory cytokine profile characterized by IL-10 production (55, 56) and enriched with genes of TGF-β signaling in CL skin (48). Together, these findings demonstrate that γδ T cells expand in response to the immunosuppressive environment created during *Leishmania* infection, adopting a dual role capable of modulating inflammation while maintaining immune balance. Their production of both regulatory and inflammatory cytokines highlights their plasticity and suggests that γδ T cells may act as key intermediates between immune activation and suppression in the course of infection.

Relatedly, a study detected CD4^+^CD25^+^ regulatory T cells producing IL-10, which suppress the proliferation of effector CD4^+^CD25^-^ cells and the production of cytokines IL-2 and IFN-γ during *L. amazonensis* infection (57). This reinforces the idea that multiple regulatory mechanisms converge to dampen effector T cell activity during *L. amazonensis* infection. This parasitic infection also leads to reduced expression of CD80 and CD1a, as well as decreased secretion of IL-6 and IFN-γ. This suggests that *L. amazonensis* may indirectly induce the suppression of T cell immune responses by reducing the signals necessary for the activation and proliferation of these lymphocytes in antigen-presenting cells, particularly dendritic cells (58). Consequently, the parasite not only suppresses effector responses but also impairs antigen presentation, further weakening host defense.

*L. amazonensis* and *L. braziliensis* infection is also capable of inducing the expression of exhaustion markers such as Programmed Cell Death Protein (PD)-1 on CD4^+^ and CD8^+^ T cells, as well as PD-L1 on dendritic cells. Treatment with anti-PD1 and anti-PD-L1 restores the ability of T lymphocytes to produce IFN-γ and reduces levels of IL-4 and TGF-β (59, 60). *L. amazonensis* infection also induces the expression of PD-L1 in human and murine neutrophils. The co-culture of infected neutrophils with CD8^+^ T cells inhibit IFN-γ production through a PD-1 and PD-L1-dependent mechanism. Notably, in this study, *in vitro* infection of human neutrophils by *L. braziliensis* also induced PD-L1 expression, and PD-L1^+^ neutrophils were detected in lesions of patients with cutaneous leishmaniasis (61). These findings highlight the involvement of immune checkpoint pathways in maintaining T cell exhaustion and suggest potential targets for therapeutic intervention.

Activation of Toll Like Receptor (TLR)-9 by the *L. amazonensis* PH8 strain induces the expression of CD200, an immunosuppressive glycoprotein that negatively regulates Nitric Oxide (NO) production (62). This likely contributes to the strong Th2 response observed in patients. However, this response appears to depend on the strain; infection with the *L. amazonensis* Josefa strain in TLR9-deficient animals leads to greater tissue damage and parasitic load, along with reduced IFN-γ at the infection site (63). Therefore, the interaction between parasite strain and host innate signaling pathways may determine the balance between protection and pathology.

In DCL, there is also a high release of TGF-β and IL-10 by macrophages and T and B lymphocytes. These cytokines have immunosuppressive and pro-apoptotic properties, with their levels increased during *L. amazonensis* infection (34, 64–67) which may lead to the suppression of CD4^+^ T cell responses. The contribution of maintaining this regulatory environment is also characterized by a high presence of Foxp3^+^ regulatory T cells in lesions of patients with DCL, compared to all other clinical forms of cutaneous leishmaniasis (68). Thus, the dominance of regulatory T cells and suppressive cytokines represents a hallmark of diffuse cutaneous leishmaniasis, underpinning its severe and persistent clinical presentation.

Overall, these findings indicate that γδ T cells play a multifaceted role during *Leishmania* infection, acting as both regulators and potential effectors of the immune response. Their expansion under conditions of αβ T cell suppression, together with their capacity to produce both pro- and anti-inflammatory cytokines, highlights their adaptability to the immunological context established by the parasite. Moreover, the predominance of regulatory cytokines such as IL-10 and TGF-β associated with γδ T cells suggests that these lymphocytes may contribute to the persistence of infection by reinforcing the immunosuppressive environment observed in cutaneous and diffuse forms of leishmaniasis. Therefore, understanding the molecular and cellular mechanisms that drive γδ T cell activation, plasticity, and migration during *Leishmania* infection could provide crucial insights into host–parasite interactions and identify potential targets for therapeutic intervention.

## TCR-δ usage in human leishmaniasis

A detailed analysis of TCRδ gene usage in *L. braziliensis*-infected patients (37) revealed that Vδ1Jδ1- and Vδ2Jδ1-bearing cells were the predominant infiltrating γδ T cells in dermal granulomas, with Vδ2 cells being the most common. In contrast, Vδ1Jδ1 cells were more prevalent in the epidermis. This distribution suggests a compartmentalized organization of γδ T cell subsets within the skin, where Vδ2 cells may dominate inflammatory sites in the dermis, while Vδ1 cells preferentially localize to the epidermis, possibly contributing to epithelial integrity and immune surveillance. Similar results were observed in the dermis and epidermis of lesions in healthy donors and patients with oriental cutaneous leishmaniasis (OCL), generally caused by *L. tropica* (69, 70). The recurrence of this pattern across different *Leishmania* species reinforces the idea that γδ T cell subset localization can be a conserved feature of skin immunity. Additionally, Vδ2Jδ3 rearrangements were present in PBMCs but absent in the lesions of *L. braziliensis*-infected patients (37), however, was found in the lesion of healthy donors and patients with OCL, indicating a limitation of the previous study’s technique in detecting Vδ2Jδ3 rearrangements in the skin or a selective localization of the Vδ2Jδ1 subpopulation to the lesions. This selective presence may reflect the recruitment of distinct γδ T cell clones to specific tissue compartments depending on the nature of the infection and antigen exposure. In general, no rearrangements were found for Vδ1/Jδ2, Vδ1/Jδ3 and Vδ2/Jδ2 (37, 70). Altogether, these findings point to a restricted pattern of γδ T cell receptor usage during *L. braziliensis* infection, suggesting that only certain subsets are actively engaged in the local immune response.

**Figure 1:**
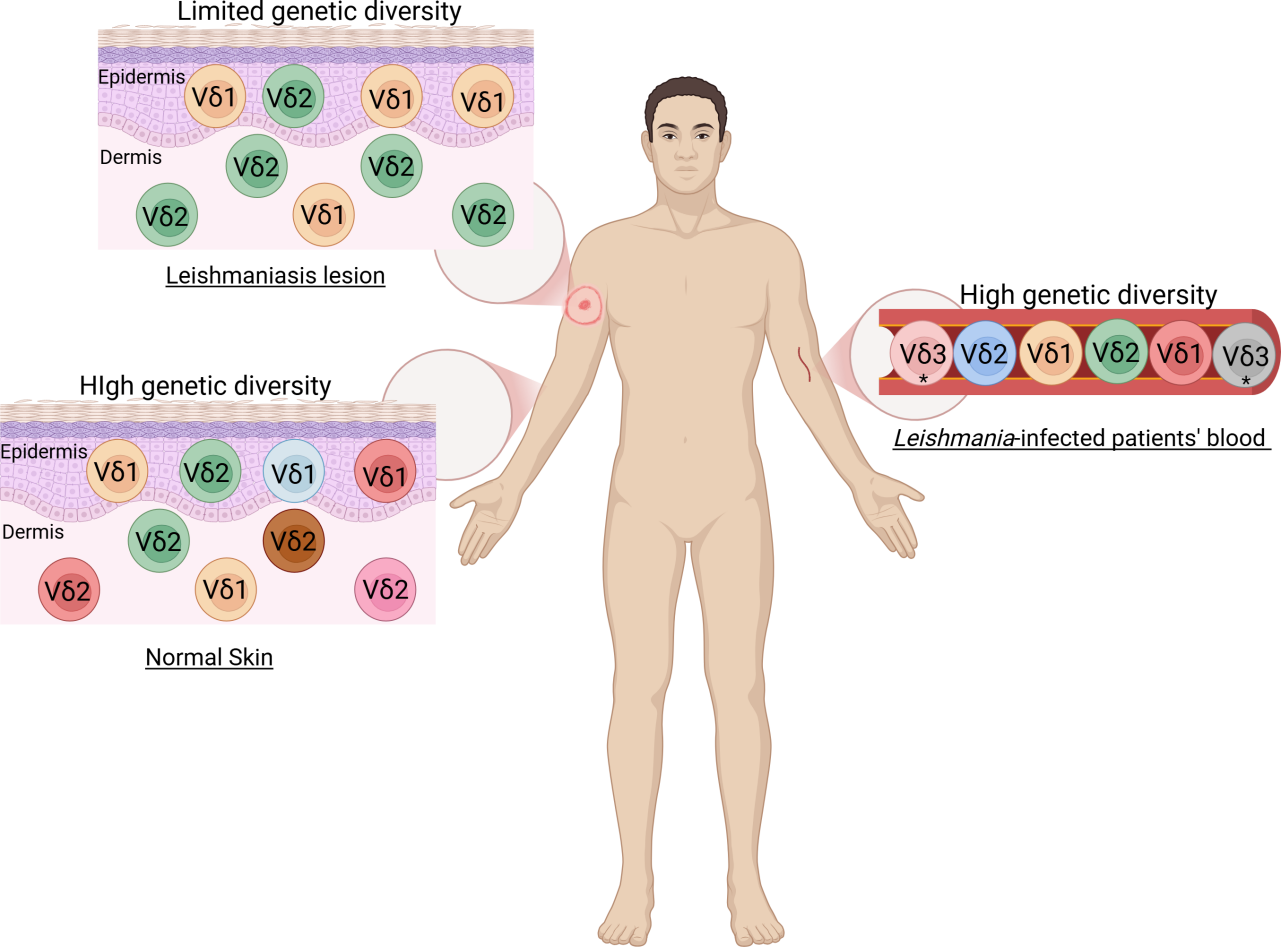
Distribution of γδ T cells in human leishmaniasis. Different colors of the same population represent high diversity in normal skin and blood of patients, unlike in leishmaniasis lesions, which show low diversity. *The Vδ3 population was found in a patient who exhibited other clinical manifestations in addition to leishmaniasis.

Besides, nucleotide sequence analysis of the Vδ-Jδ junctions in the lesions in *L. braziliensis*-infected patients and patients with OCL showed limited genetic diversity, with unique sequences appearing multiple times (37, 70), unlike the extensive diversity seen in the *L. braziliensis*-infected patients’ blood and normal skin (37, 70) (**Figure 1**). This contrast between circulating and tissue-resident γδ T cells suggests a local clonal restriction driven by antigenic selection within the lesion microenvironment. Furthermore, in *L. braziliensis*-infected patients’ lesions the limited diversity of Vδ1 and Vδ2 populations was notable in 25-μm sections, it was less apparent in 100-μm samples. Unique sequences were predominantly identified in adjoining sections, however, the main sequence of one area appeared as a minor sequence in an adjacent area supporting the idea that clonal selection and oligoclonal expansion of γδ T cells occur in specific microanatomic environments with restricted dispersal throughout the tissue (37). Limited diversity suggests that, rather than a broad range of γδ T cells responding to an antigen (71, 72), a restricted number of clones (γδ T cells with the same receptor) are expanding in response to a specific stimulus. Such focused expansion indicates that specific subsets of γδ T cells recognize particular antigens or stress-induced ligands associated with *Leishmania* infection. This occurs because, after activation by an antigen, some γδ T cells can proliferate more than others, resulting in an increase in the copies of certain clones, as seen in cytomegalovirus (CMV) infection and common variable immunodeficiency (CVID) (73, 74). Thus, the presence of reduced diversity of γδ T cell receptors indicates a targeted response to a specific antigen, characterizing oligoclonal expansion. A predominance of Vδ1-N-Dδ3-N-Jδ1 and Vδ2-N-Dδ3-N-Jδ1 sequences in the lesions and the examination of the deduced amino acid sequences based on Vδ2-Vδ1 junctional nucleotide sequences from normal skin, OCL patients, and *L. braziliensis*-infected patients revealed similar amino acid insertions, which can be attributed to the consistent incorporation of Dδ3 gene segments (37, 70). Taken together, these findings strongly support that γδ T cells undergo antigen-driven expansion within *Leishmania*-infected skin, reflecting a specialized immune adaptation that contributes to the modulation of local immune responses during parasite persistence.

## γδ T cells in human leishmaniasis treatment outcomes

There are clearly differences in the responses mediated by *L. braziliensis* and *L. amazonensis*. For instance, the disease caused by *L. amazonensis* shows a more intense suppression of Delayed-type hypersensitivity (DTH) responses compared to that caused by *L. braziliensis*. This directly impacts the response to antimonial therapy, as patients with *L. amazonensis* disease require double the dosage used for treating patients with *L. braziliensis* disease (75). Most patients with DCL, particularly children under five, endure severe and sometimes highly mutilating clinical conditions for many years, leading to chronic disease. This is partly because available treatments (pentavalent antimony, amphotericin B, pentamidine, and miltefosine) do not alter the Th2-skewed profile characteristic of these patients (76). As a result, relapses are common among patients with DCL (77–79).

In this context, the expansion of γδ T cells from PBMCs of *L. major*-infected patients with active cutaneous leishmaniasis, both with and without glucantime therapy, was investigated (38). Significantly more γδ T cells were observed in untreated patients (~15%) compared to those treated with glucantime (~4%) and healthy donors (~5%), representing an approximate 11% absolute decrease following treatment. Conversely, a significant increase in αβ T cells was noted in glucantime-treated patients (~62%) compared to untreated patients (~55%), an approximate 6–7% absolute increase, and healthy donors (~55%). This suggests a compensatory mechanism in which γδ T cells expand preferentially when αβ T cell responses are suppressed or less active. Similar results were found in *L. infantum*- infected dogs treated with amphotericin B (80) and *L.donovani*-infected patients treated with glucantime (81). However, in patients infected with *L. major* and treated with sodium stibogluconate (Pentostam, SSG), although an increase in γδ T cells was observed compared to αβ T cells in post-treatment patients, no difference in γδ T cells was found when compared to healthy individuals or patients before and after treatment (82). This suggests that the type of treatment chosen may influence the expansion of γδ T cells during *Leishmania* infection. Patients from an area endemic for *L. braziliensis* (55), despite not mentioning the treatment that the infected patients received, in non-infected individuals, the DN αβ cells range from 0.92% to 2.30%, while the DN γδ cells range from 2.12% to 6.83%. In contrast, in patients with CL, the DN αβ cells increase to between 1.87% and 5.28%, whereas the DN γδ cells show a significant reduction, ranging from 0.33% to 1.75%. Furthermore, the DN αβ cells account for 72% of the DN T cells in patients with CL. This change suggests an alteration in the immune response of the patients, with a notable predominance of DN αβ T cells over DN γδ T cells during the infection. Additionally, compared to untreated mice, the percentage of γδ T cells was reduced by 50% in the spleens of glucantime-treated BALB/c mice. These data indicate a preference for the proliferation of γδ T cells in the absence of αβ T cells. Moreover, this could imply that when glucantime is present, the parasite load diminishes, leading to a reduced necessity for the expansion of γδ T cells. Besides, there does not appear to be a significant relationship regarding the frequency of γδ T cells in lesions of *L. braziliensis*-infected patients treated or not with glucantime, but this relationship needs further evaluation (47). Taken together, these observations highlight the context-dependent nature of γδ T cell expansion, influenced by treatment, parasite species, and host immune status.

The relationship of γδ T cells in active disease or treated patients is more complex because analyzing a sample of PBMCs from 40 patients (83), γδ T cells were found in both active and cured leishmaniasis patients. For the cured visceral group, the average was 7.20%; for the active or recently treated cutaneous group, the average was 8.1% and for the mucosal group, the average was 7.52% in comparison to health donors. This indicates that γδ T cells can persist in circulation even after clinical cure, suggesting a potential role in long-term immune surveillance or memory-like functions. In some leishmaniasis patients who were cured or recently treated, levels of circulating γδ T cells remained high. In three cutaneous patients, blood samples taken before and 3 to 10 weeks after treatment showed one patient’s γδ T cell percentage increased from 3% to 8%, while the other two maintained levels of 10% to 11%, with a slight rise to 12%. Among the tested patients, all had significant percentages of circulating γδ T cells co-expressing CD8 (20% to 40%), the other γδ T cells were CD4^−^, CD8^−^, and none of the patients co-expressed CD4 with the γδ TCR (83). These data suggest that γδ T cells, particularly those co-expressing CD8, may represent a distinct functional subset capable of persistent activity after infection, independent of αβ T cell dynamics.

## γδ T cells and human leishmaniasis duration

Although γδ T cells were initially associated with the early stages of granuloma formation in *L. braziliensis* (37, 45) and *L. tropica* (69) infection, these cells were found in varying amounts in lesions of cutaneous, mucosal, and disseminated leishmaniasis, regardless of the clinical characteristics of the lesions, including duration and parasite load (39, 55, 69). This variability suggests that γδ T cell recruitment to lesions is not strictly dependent on disease stage or parasite density, but may instead reflect individual immune dynamics. However, it is important to emphasize that an analysis of each patient is necessary to correlate the presence of γδ T cells with the outcome of leishmaniasis.

Among the 11 cases of OCL (69), an increased percentage of γδ T cells (15-20% of CD3^+^ cells) was observed in three patients (Patients 2, 3, and 8). Of these, Patients 2 and 8 presented early lesions with durations of 1 and 1.5 months, respectively, while Patient 3 had a lesion that lasted 8 months. However, the other 8 patients with early lesions between 1 and 3 months had only between <1% and 5% of γδ T cells. These findings indicate that the accumulation of γδ T cells in lesions does not follow a linear relationship with lesion age, suggesting that additional factors, such as local cytokine milieu or genetic background, may regulate their recruitment or retention. Despite these differences, no correlation was found between the frequency of γδ T cells and duration of the lesion with the number of parasites (69). Therefore, γδ T cell abundance alone may not be a reliable marker of parasite control, but rather a reflection of ongoing immune modulation within the lesion.

Additionally, among 13 patients—5 with VL, 1 with ML, and 8 with CL—there were no differences in the frequency of γδ T cells in the PBMCs of these patients (83). This lack of systemic variation across clinical forms reinforces the notion that γδ T cell responses are predominantly localized and context-dependent within infected tissues. Among 4 patients infected with *L. donovani*, Patient 3 exhibited higher expression of CD25 and HLA-DR on γδ T cells present in PBMCs. This phenotype suggests an activated or antigen-experienced state, possibly driven by persistent inflammatory cues. Indeed, there appears to be an MHC-dependent immune response induced by inflammation during CMV and *Leishmania* infection in γδ T cells (48, 84). In contrast, γδ T cells from Patient 2 showed lower expression of CD38, while γδ T cells from Patients 3 and 4 displayed reduced expression of CD71 compared to Patients 1 and 2 (40). Such heterogeneous expression of activation markers further underscores the functional diversity of γδ T cells in human leishmaniasis, reflecting distinct activation thresholds and metabolic states across individuals. Besides, the reduced expression of CD38 and CD71 on γδ T cells may reflect distinct functional adaptations rather than a uniform decrease in activation. CD38 downregulation could indicate a metabolically less active or quiescent state, consistent with studies linking CD38^high^ expressing T cells to strong immune activation in visceral leishmaniasis (85–87). Conversely, reduced CD71 expression suggests limited proliferative capacity or altered iron uptake, possibly influenced by parasite-induced changes in host iron metabolism and erythropoiesis (88, 89). Together, these findings point to a functionally restrained or metabolically reprogrammed γδ T-cell profile, potentially shaped by the immunometabolic environment of *Leishmania* infection.

## Human visceral leishmaniasis

In visceral leishmaniasis, specifically in PBMCs from *L. donovani*-infected patients, there is a marked expansion of γδ T cells within the total CD3^+^ population, from approximately 8% in healthy donors to 44% in infected individuals, accompanied by a corresponding decrease in αβ T cells, from 92% in healthy controls to 53% in patients. This pattern was also observed within the CD4^+^CD8^−^ and CD4^−^CD8^+^ subsets, with only minor variations detected in the CD4^−^CD8^−^ population (40). Although in cutaneous leishmaniasis only the co-expression of TCRγδ and CD8^+^ was observed while a few proportions of γδ T cells are CD4^-^CD8^-^ (83), in VL a large percentage of γδ T cells expressing CD4^+^CD8^-^ was found in *L. donovani*-infected patients (40). Additionally, a small proportion of γδ T cells expressing CD4^-^CD8^+^ (83) and CD4^-^CD8^-^ was identified in both *L. donovani*-infected patients and controls. It was observed that *L. donovani*-infected patients exhibited an increase in CD4^+^CD29^+^ expressing γδ T cell subsets, indicating a significant role for γδ T cells. Specifically, while no differences in TCRαβ and TCRγδ expression were noted in the CD4^+^CD45R^+^ subset, the CD4^+^CD29^+^ subset from VL patients showed a higher percentage of TCRγδ expression compared to TCRαβ. The data confirmed that the CD4^+^CD29^+^ expressing γδ T cells are the primary contributors to these effects (40). These findings underscore the importance of CD4^+^CD29^+^ expressing γδ T cells in the immune response of VL patients. Human CD4^+^Vδ1^+^ γδ T-cell subpopulation can transdifferentiate into functional αβ T cells both ex vivo and *in vivo*, resembling thymic T-cell development. This process involves the reorganization of the γδTCR into the αβTCR and is detectable in inflamed tissues (90). Such plasticity further supports the hypothesis that γδ T cells play a dynamic role in shaping adaptive immunity under inflammatory or infectious conditions, such as those induced by *Leishmania* infection. These results highlight that γδ T cells, particularly the CD4^+^CD29^+^ subset, undergo selective expansion and phenotypic modulation during visceral leishmaniasis, suggesting their active participation in disease-associated immune regulation.

Similar results regarding the expansion of γδ T cells were also observed when PBMCs from patients infected with the *L. donovani* AG83 strain were stimulated with purified amastigotes prepared from the spleens of hamsters infected with the same strain, compared to PBMCs that were not exposed to the parasite and when the PBMCs were stimulated with *L. donovani* HK (41). Additionally, no significant differences were observed in αβ T cells, with only a 1.1-fold decrease compared to a notable 3.6-fold increase in γδ T cells. It was observed that approximately 30% of the γδ^+^ T cells expressed intracellular *L. donovani* antigens. However, although not shown, the authors reported that γδ-enriched T cells from PBMCs did not contain intracellular amastigotes (41). These observations reinforce the notion that γδ T-cell activation in VL is driven predominantly by antigenic stimulation rather than direct infection, pointing to their role as rapid responders to *Leishmania*-derived signals.

## γδ T cell-mediated immunity in human leishmaniasis

Initially, Modlin and colleagues (45) speculated that the accumulation of antigen-specific TCR γδ cells in significant numbers during immunological reactions and lesions characterized by active granuloma formation suggested that γδ T cells, or the products they secrete, might contribute to the development of such lesions. They then analyzed that a significant bone marrow-derived macrophages (BMDM) aggregation and cell division only occur when CD3-activated γδ T cell supernatants derived from CL lesions of *L. braziliensis*-infected patients were combined with Granulocyte-macrophage colony-stimulating factor (GM-CSF) in comparison to unactivated γδ T cell supernatants in the presence of GM-CSF. This suggests that cytokines-secreted by γδ T cell upon antigen stimulation promote macrophage adhesion, aggregation, and proliferation and can contribute to granuloma formation. Regarding the ability of γδ T cells to be stimulated by *Leishmania* antigens, Pfeffer K and colleagues (91) demonstrated that γδ T cells as well as αβ T cells exposed to *Leishmania*, leishmanial antigen preparations or disrupted *Leishmania* (SLA), show reduced proliferation (**Table 1**). These findings indicate that some leishmanial antigens cannot trigger a significant immune response in unprimed human donors.

**Table 1:** TCR δ gene usage, sublocalization and markers of γδ T cells in human leishmaniasis.

Strain	Markers/Co-expression	Disease	Site	TCR δ gene usage	Ref.
*L. braziliensis* (strain N/D)	N/D	CL and ML	Skin PBMCs	Vδ1	(45)
Endemic area for *L. braziliensis* (strain N/D)	N/D	CL	Dermis epidermis PBMCs	Dermis Vδ1Jδ1 < Vδ2 Jδ1 Epidermis Vδ1Jδ1 > Vδ2 Jδ1 PBMCs Vδ1Jδ1, Vδ2Jδ1 and Vδ2Jδ3	(37)
Endemic area for *L. braziliensis* (strain N/D)	N/D	CL	Skin	Vδ1	(47)
Endemic area for *L. braziliensis* (strain N/D)	N/D	CL, DCL and ML	Skin	Vδ1	(39)
Endemic area for *L.braziliensis* (strain N/D)	CD25Low CD28 CD69 CD45RO	CL	PBMCs	N/D	(55)
*L. Tropica* (strain N/D)	N/D	CL	Dermis epidermis	Dermis Vδ1 < Vδ2 Epidermis Vδ1 > Vδ2	(69)
*L. Tropica* (strain N/D)	N/D	CL	Dermis epidermis	Vδ1Jδ1 Vδ2Jδ1 Vδ2Jδ3	(70)
Endemic area for Leishmaniasis (strain N/D)	CD8^+^ CD4-CD8-	CL and VL	PBMCs	Vδ1	(83)
Institute of Biomedicine (strain N/D)	N/D	CL, DCL and ML	Skin	Vδ1	(46)
*L. major* (strain N/D)	N/D		PBMCs	Vδ1	(38)
*L. donovani* (strain N/D)	CD4^+^CD8^−^ CD4^+^CD8^−^CD45R^+^ CD4^+^CD8^−^CD29^+^ CD4^-^CD8^+^ CD4^-^CD8^-^ CD25 CD38 CD71 HLA-DR	VL	PBMCs	Vδ1	(40)

Besides, T cell lines generated from PBMCs of cutaneous and mucosal patients, stimulated with *L. amazonensis* or *L. braziliensis* promastigote lysate, revealed high percentages of γδ T cells (25% to 60%). However, in antigen-specific T cell lines from normal donors stimulated with gp63 and gp42, the percentages of γδ T cells did not increase. Similar results were observed with γδ-enriched T cells populations obtained through cell sorting, which were cultured with APCs and *L. amazonensis* promastigote lysate. In this case, αβ T cell proliferation also occurred (**Table 1**). These data suggest that γδ T cells recognized and responded to *Leishmania* antigens such as lipids other than gp63 or gp42. Additionally, patients with DCL exhibit a negative DTH skin test, indicating suppression of the Th1 response, as well as an absence of lymphocyte proliferative responses to *Leishmania* antigens (92, 93). Anergy is limited to cellular immune responses specific to *Leishmania* antigens, as responses of γδ T cells to unrelated antigens remain intact (45, 91). When γδ-enriched T cells from PMBCs of mucosal patients were stimulated with Heat Shock Protein 70 (Hsp70) of *L. chagasi* they had a greater proliferation, however, with a small proliferation with *L. braziliensis* promastigote lysate (**Table 1**). Altogether, these findings demonstrate that γδ T cells display selective antigen recognition in leishmaniasis, responding preferentially to conserved or stress-related molecules such as Hsp70, rather than to major surface antigens like gp63.

In VL, it was observed that γδ T cells from PBMCs of *L. donovani*-infected patients exhibit a higher activation profile compared to αβ T cells, as indicated by the expression of CD25^+^, CD38^+^, CD71^+^, and HLA-DR^+^ (40). Moreover, in healthy donor, γδ T cells showed a lower response to stimuli *in vitro* compared to αβ T cells. In *L. donovani*-infected patients, the response of γδ T cells to PHA and anti-CD3 was also reduced. However, these γδ T cells exhibited a significantly elevated response to stimulation with recombinant human IL-2 (rhIL-2), indicating their heightened sensitivity to this cytokine in VL patients (40). Regarding cytokine production, In healthy donors, the supernatants from αβ T cells exhibited higher levels of IL-2, as well as factors associated with B cell growth (BCGF) and differentiation (BCDF), compared to those from γδ T cells, as measured by the CTLL (IL-2-dependent murine cell line) proliferation assay, B cell proliferation, and the production of Immunoglobulin (Ig) M and IgG, respectively. In patients infected with *L. donovani*, the supernatants from αβ T cells contained lower levels of these factors compared to healthy donors. Interestingly, while γδ T cells from VL patients also produced low levels of IL-2, the supernatants from these cells showed significantly increased amounts of both BCGF and BCDF measured by B cell proliferation and the production of IgM and IgG (40). These data demonstrate that patients with VL, γδ T cells can work with dual role by exhibiting an impaired cell-mediated immune response due to their reduced production of IL-2. On the other hand, their increased ability to produce BCGF and BCDF could enable them to mount a significantly enhanced humoral immune response (40). γδ T cells, unlike αβ T cells, contribute to various aspects of humoral immunity, including the maturation, activation, and differentiation of B cells, as well as antibody production and class switching in different contexts (94–96). Therefore, further investigations are needed to assess the impact of γδ T cells on the humoral response in leishmaniasis.

Interestingly, a case report observed the immune response in the blood of a patient with thymoma, chronic visceral leishmaniasis, and myasthenia gravis (97). This patient exhibited an increase in circulating γδ T cells (~30%), with approximately 60% of the cells expressing Vδ1, 22% Vδ2, and 18% Vδ3. Regarding the expression of the γ TCR variable chain, although data were not shown, an expansion was observed with 20% of cells expressing Vγ9, 24% Vγ8, 20% Vγ5, and 28% Vγ4. These cells displayed a naïve phenotype when compared to healthy donors (97). The γδ T cells from this patient were unable to proliferate upon re-stimulation *in vitro* with *L. donovani* antigens. Furthermore, the TCRγδ complex in the patient’s PBMC preparations contained less ζ protein (CD247) than the controls, relative to the amount of CD3ϵ protein, indicating a modification of the TCR/CD3 complex in these patients (97). However, since there were no differences in CD247 levels between naïve and memory T cells in healthy donors, the reduction of CD247 within the TCR complexes of the patient’s naïve T cells may be a consequence of thymoma (97). Therefore, further studies are needed to assess potential alterations in the TCRγδ/CD3 complex triggered by *Leishmania* infection. Ferraz et al. (98) highlighted that CD3^+^CD4^−^CD8^−^ T cells and NKT cells are the main cytotoxic CD107a^+^ cells in lesions of cutaneous leishmaniasis caused by *L. braziliensis*. The frequency of cytotoxic CD107a^+^ cells was positively correlated with granzyme B production at the lesion site. Furthermore, lesion size was positively correlated with granzyme B production, the frequency of CD107a^+^ cells, and the expression of IFN-γ and TNF-α. However, further studies are needed to assess whether γδ T cells predominate in this CD4^−^CD8^−^ population. Clarifying this association could help define whether γδ T cells represent a key cytotoxic subset contributing to tissue damage and parasite control in cutaneous leishmaniasis.

## γδ T cells in experimental leishmaniasis

### L. major

**Figure 2:**
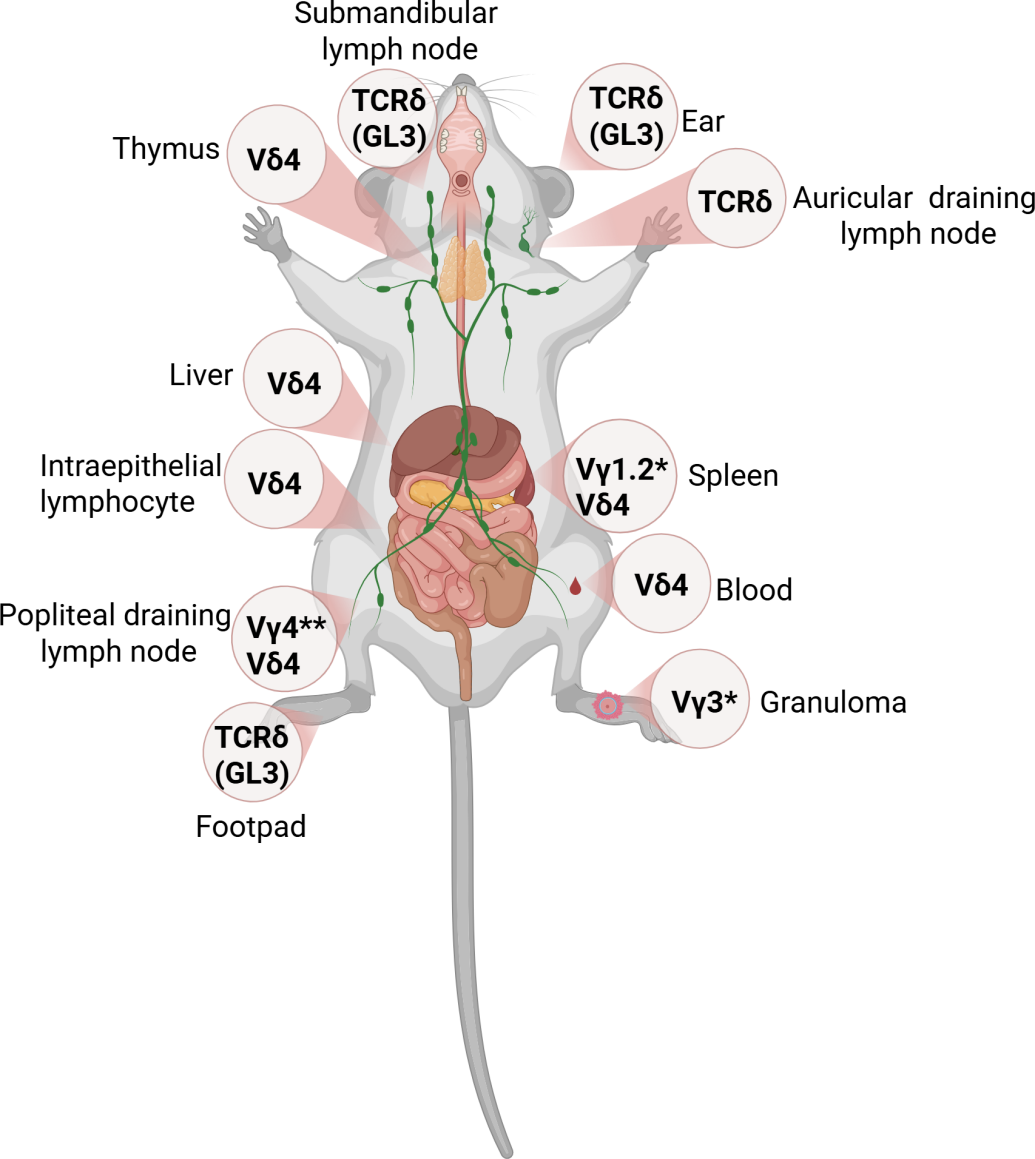
Localization of γδ T cells in experimental models. Representation of γδ T cells across different tissues, with nomenclature of the γ subunit according to Garman (*) and Tonegawa (**).

The first study evaluating γδ T cell responses in experimental *Leishmania* infection (99) showed that γδ T cells do not significantly increase in frequency in the popliteal draining lymph node or the spleen of BALB/c and C57BL/6 mice infected (**Figure 2**) with *L. major* (strain: MHOM/IL/81/FE/BNI). It was observed that γδ T cells accounted for less than 0.4% in the lymph node and less than 0.9% in the spleen during the first 49 days post-infection. These findings indicate that γδ T cells do not play a critical role in the systemic progression of *L. major* infection, at least for this strain and experimental setting. However, in the context of infection with the *L. major* LV39 strain (MRHO/SU/59/P) (100), BALB/c mice exhibited a less pronounced expansion in the draining lymph node starting at day 18 post-infection, while a significant increase was observed in the spleen, where approximately 35% of the CD3^+^ cells were γδ T cells. Conversely, in CBA/J mice, there was no notable expansion in the draining lymph node; however, the response of γδ T cells peaked around day 30, constituting 12% of the CD3^+^ population at that time. At 40 days of infection, γδ T cells in the spleen of infected BALB/c mice revealed an increased proportion of γδ T cells expressing the CD8α chain, while neither the CD8β chain nor the CD4 molecule was detected (100). Subsequently, the number of γδ T cells declined alongside the spontaneous resolution of the lesions (100). Together, these results suggest that the kinetics and magnitude of γδ T cell expansion depend on the host genetic background and the strain of *L. major*, indicating a context-dependent role of these cells in infection outcome.

In relation to the number of these cells, a significant increase was observed in the populations of αβ and γδ T cells 18 days after *L. major* infection (100). In BALB/c mice, γδ T cells showed remarkable expansion, particularly in the lymph nodes, although there was a lesser expansion in the spleen. In CBA/J mice, the expansion of γδ T cells was also notable, albeit to a lesser extent. These results reinforce the notion that γδ T cell proliferation represents an active immune response to infection rather than a passive reflection of αβ T cell migration. This data also reveals a different dynamic between the absolute numbers and the percentages of γδ T cells (100). Therefore, early γδ T cell expansion may represent a transient but functionally relevant component of the immune response to *L. major*.

Next, the impact of treatment using anti-γδ TCR antibody was analyzed. The treatment with anti-γδ TCR GL3 in *L. major*-infected BALB/c mice leads to exacerbation of lesion development and increased parasite load compared to control mice (**Table 2**). Although the data were not shown, no effect of the administration of GL3 on the levels of CD4^+^ or CD8^+^ cells was observed in *L. major*-infected BALB/c mice. In *L. major*-infected CBA/J mice, the treatment also worsened the lesions, resulting in a delayed healing process compared to control mice (100). These findings confirm that γδ T cells contribute to parasite control and lesion resolution, highlighting their protective role during infection.

**Table 2:** Activation of γδ T cells in human leishmaniasis.

Condition/Strain	Stimulus	Proliferation	Culture added	Production	Ref.
*L. major* (MHOM/IL/81/FE/BNI)	Live *L. major* rhIL-2	Poorly			(91)
*L. major* (MHOM/IL/81/FE/BNI)	LPG rhIL-2	Poorly			(91)
*L. major* (MHOM/IL/81/FE/BNI)	SLA rhIL-2	Poorly			(91)
PBMCs of cutaneous and mucosal patients (Strain N/D)	*L. amazonensis* promastigote (IFLA/BR/67/PH8) or *L. braziliensis* promastigote (MHOM/BR/OO/LTB67S) lysate rHIL-2	Yes	APC		(83)
PBMCs of cutaneous and mucosal patients (Strain N/D)	gp63 and gp42 of *L. amazonensis* rhIL-2	Poorly			(83)
γδ-enriched T cell of PBMCs of cutaneous patients (Strain N/D)	*L. amazonensis* promastigote (IFLA/BR/67/PH8) lysate	Yes	APC		(83)
γδ-enriched T cell of PBMCs of mucosal patients (Strain N/D)	*L.* chagasi promastigote (MHOM/BR/82/BA-2)	Poorly	APC		(83)
γδ-enriched T cell of PBMCs of mucosal patients (Strain N/D)	HSP 70 *L. chagasi*	Yes	APC		(83)
γδ-enriched T cell of PBMCs of *L. donovani* infected patients (Strain N/D)	PHA Anti-CD3 rhIL-2	PHA - Poorly Anti-CD3 – Poorly rHIL-2 - Yes		IL-2 BCGF BCDF	(40)
PBMCs of healthy donor	Live and HK *L. donovani* amastigotes (Strain AG83) rHIL-2	Live – Yes HK - Poorly			(41)
PBMCs of healthy donor	Live *L. aethiopica* promastigotes Heat Killed Live *L. aethiopica* promastigotes Freeze thawing Killed *L. aethiopica* promastigotes	Poorly			(174)

After staining with propidium iodide (PI), sorted γδ T cell populations isolated from the spleens of chronically infected BALB/c mice contained more cycling cells than similarly sorted CD4^+^ T cells. These data suggest that γδ T cells are more active and proliferative during the *L. major* infection (101), indicating their potential role in the immune response against the parasite. The authors also reported, although they did not present the data, that γδ T cells from *L. major*-infected BALB/c mice exhibit restricted Vγ gene usage (100), thus supporting the oligoclonality of the γδ T lymphocyte response, as observed in patients (37, 69). Confirming this hypothesis through the analysis of Vδ gene usage, a significant increase in γδ T cells expressing Vδ4 was observed in the spleen of BALB/c mice infected with *L. major* (101). This increase was also noted in the lymph nodes, liver, and blood, but not in the thymus or the IEL compartment, demonstrating an oligoclonal expansion of these T cells in response to the infection. Although the data is not shown, it was mentioned that the Vγ gene usage of Vδ4, examined by PCR analysis, highlighted the use of the Vγ1.2 (Garman) or Vγ2 (Tonegawa) gene segment (101). FACS analysis of activation marker expression on γδ T cells in the spleen of chronically infected BALB/c mice revealed increased levels of LFA-1, CD25, CD44, 4F2, CD28, and the heat-stable antigen (HSA), while expression of Thy-1 and CD5 was decreased. In contrast, analysis of the same markers on the αβ T cell population showed no significant differences between cells from normal and infected animals (101). Together, these results indicate that γδ T cells undergo a selective and antigen-driven expansion during infection, accompanied by an activated phenotype, suggesting that these cells may participate in both the regulation and effector phases of the immune response.

Expanding γδ T cells in *L. major*-infected BALB/c mice, observed from 2 weeks to 3 months post-infection (100), suggests that this response may depend on thymus-derived γδ T cells or the presence of αβ T cells, rather than being solely triggered by parasite antigens. In fact, BALB/c mice infected with *L. major* exhibit a greater expansion of γδ T cells in the spleen compared to both BALB/c and Swiss nu/nu mice. Notably, there is a more pronounced expansion of Vδ4 T cells in comparison to total γδ T cells, indicating a selective expansion of this subset during *L. major* infection in susceptible BALB/c mice (102). This expansion was not observed in TCRαβ-deficient mice. Treatment with anti-CD4, but not anti-CD8, resulted in a significant reduction (~60%) of Vδ4 T cells in the spleen of *L. major*-infected BALB/c mice, supporting the crucial role of CD4 T cells in the expansion of γδ T cells during *L. major* infection in susceptible BALB/c mice (102). Interestingly, in genetically resistant IFN-γ receptor-deficient 129 mice infected with *L. major*, γδ T cell expansion was absent, which may be explained by the continued IFN-γ production in these mice, despite the lack of a Th2 response (103). The treatment of non-infected BALB/c mice with anti-IgD, which is known to induce a Th2 phenotype, increased the expansion of Vδ4 T cells, similar to the expansion observed in untreated infected mice. Although to a lesser extent, this treatment also elevated the frequency of Vδ4 T cells in CBA mice and following *Nippostrongylus brasiliensis* infection in C57BL/6 mice (102). These findings suggest that the Th2-type CD4 cell effect is a general phenomenon, not restricted to *L. major* infection, and also reflect the influence of genetic background on the dynamics of γδ T cell expansion during *L. major* infection. These observations reinforce that γδ T cell expansion is strongly influenced by the host’s genetic background and by CD4^+^ T cell–dependent cytokine environments, particularly those associated with Th2-type responses. The data also suggest that γδ T cells respond to broader immunological cues beyond direct parasite recognition.

A large number of *L. major* promastigotes were recovered from the macrophages in regional lymph nodes of both anti-TCRαβ and anti-CD4 treated C57BL/6 mice, while only small numbers were recovered from those treated with anti-γδ or anti-CD8 antibodies (104). Furthermore, only the treatment with anti-TCRαβ and anti-CD4 antibodies was able to inhibit the expression of HSP-65 by macrophages, with no impact observed in the anti-γδ or anti-CD8 treatments. These findings demonstrate that while γδ T cells are crucial for the expression of HSP-65 by host macrophages, contributing to early protection against *Toxoplasma gondii* (105, 106), they do not play the same role during *L. major* infection (104). This highlights that the effector functions of γδ T cells are pathogen-specific and that their protective mechanisms during *L. major* infection differ from those described in other protozoan infections.

Mou et al. described that CD3^+^CD4^−^CD8^−^ T cells restricted to MHC II produce IFN-γ, TNF, IL-17, and granzyme B in the draining lymph nodes and spleens of mice following primary and secondary *L. major* infections. Additionally, these cells exhibit memory characteristics, with rapid proliferation and cytokine production, which are impaired in the absence of CD4 T cells. DN T cells predominantly express the αβ TCR in 96% of cases and have been implicated in both primary and secondary anti-*Leishmania* immunity. However, studies that assessed the contribution of these cells through purification have sorted total DN T cells and showed that approximately 7.5% of DN T cells express the γδ TCR. Therefore, further studies are needed to properly assess the contribution of these γδ T cells during *L. major* infection in the specific strain used in this study (MHOM/80/Fredlin) (107).

**Figure 3:**
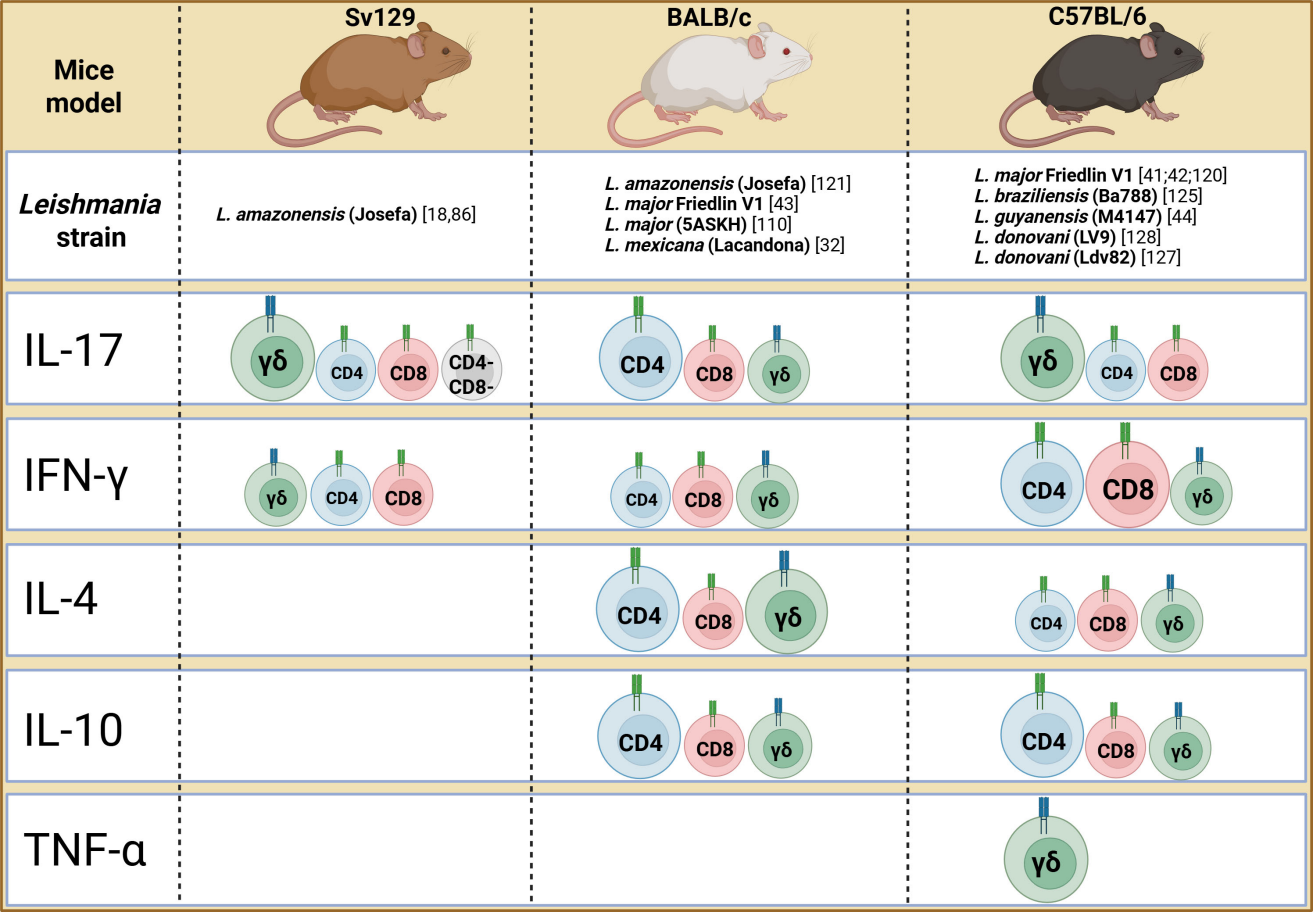
Source of cytokines by T cells in experimental models. The figure highlights the cellular sources of cytokines only in contexts where γδ T cells were evaluated. The size of the cells indicates whether the cell is the primary source (larger) or has a secondary role (smaller).

Annexin A1 signaling has been implicated in the exacerbation of *L. major* infection when associated with *L. major*-derived exosomes (108). However, no difference was observed in the frequency of γδ T cells in the lesions of WT mice, WT mice treated with exosomes, or Annexin A1-deficient mice, whether treated or not (108). Despite all these studies, Satoskar et al. (109) observed that both C57BL/6 WT mice and γδ T cell-deficient mice exhibit similar lesion profiles and parasite loads when infected with the LV39 strain. Cells from the draining lymph node of both groups of mice, stimulated with *L. major* antigens, produced similar amounts of IFN-γ, particularly at 6 weeks post-infection, demonstrating that IFN-γ produced by γδ T cells may not be crucial for parasite control in infection with this strain (109). Additionally, IL-4 production was also similar, with peak levels observed early, at 2 weeks post-infection. These findings align with previous data showing that γδ T cells are a recent source of IL-4 (**Figure 3**) during *L. major* infection (110). Taken together, these results suggest that γδ T cells are not essential for parasite control in resistant C57BL/6 mice. However, it is important to note that the role of γδ T cell deficiency should be further evaluated in other *L. major* strains.

A comparative analysis of cytokine production *in vitro* between *L. major*-infected BALB/c and C57BL/6 mice over a 3-week period showed that neither popliteal lymph node cells, spleen cells, nor hepatic mononuclear cells were able to produce IFN-γ without antigenic stimulation, with production occurring only upon stimulation with *L. major* antigens (110). However, spleen cells, and particularly hepatic mononuclear cells from BALB/c mice, were capable of producing IL-4 both with and without antigenic stimulation. In contrast, cells from C57BL/6 mice produced only low levels of IL-4 upon antigenic stimulation (110). IL-4 production by hepatic mononuclear cells in BALB/c mice was observed from the early stages of infection, between 2 to 5 weeks post-infection. CD4 T cells, but not CD8 T cells, γδ T cells, and a subset of non-characterized cells, possibly eosinophils (111), were responsible for IL-4 production during the early stages of infection (111). No increase in γδ T cells was observed in the liver throughout the infection. These observations are consistent with the notion that host–microbiota interactions shape the early immune environment that favors IL-4 induction (112). Evidence from experimental models indicates that commensal microbial antigens can condition CD4^+^ T cells toward rapid IL-4 secretion upon parasite challenge, suggesting that microbial priming contributes to the Th2 bias typical of BALB/c responses (112). In parallel, growing data support that γδ T cells are highly responsive to microbial-derived signals and can influence systemic immunity through cytokine release and modulation of antigen-presenting cells (113–116). Taken together, these findings provide a plausible framework in which γδ T cells, through their responsiveness to microbiota-associated cues, may indirectly contribute to the early IL-4 production observed during *Leishmania* infection.

In an attempt to generate a conditional deficiency of the IL-4 receptor α chain (IL-4Rα), despite efficient deletion of IL-4Rα expression on CD4 T cells which led to impaired IL-4–induced CD4^+^ T cell proliferation and Th2 differentiation that result in resistance to *L. major* infection in BALB/c mice, which are typically susceptible to the parasite, γδ T cells and non–T cells in Lck^cre^IL-4Rα^−/lox^ mice retain normal receptor expression (117). This suggests that the role of IL-4 in γδ T cells is not fully addressed by the current deletion model and highlights the need for new tools to specifically investigate IL-4 signaling in γδ T cells. However, when evaluating the role of Immediate Early Response Gene X-1 (IEX-1), a stress-inducible gene highly expressed in macrophages and T cells following *L. major* infection, it was discovered that excessive inflammation was not due to a Th2-biased immune response or a defect in Th1 polarization. Instead, the heightened inflammation was linked to increased IL-17 production by both γδ and CD4 T cells, alongside an elevated neutrophil recruitment early in the infection (118). In IEX-1-deficient 129Sv/C57BL/6 mice there was greater tissue damage and parasite load following infection. These mice exhibited a significant increase in IL-17-producing CD4 T and γδ T cells 5 days post-infection, though only IL-17 production by γδ T cells remained persistently elevated at later stages of the infection (118). This highlights a dynamic shift in the cytokine sources during *L. major* infection depending on the genetic background of the mice. While γδ T cells serve as an early primary source of IL-4 in susceptible mice (110), they become an early primary and late source of IL-17 in the resistant mouse model (118).

Indeed, regarding the production of IL-17, the pathogenic role of these cells was highlighted in high dose *L.major*-infected BALB/c mice, with CD4 T cells being the main source of IL-17, and minimal contribution from γδ T cells (43). However, in low-dose *L. major*-infected C57BL/6 mice, no impact was observed in the deficiency of IL-17A and IL-17F. In this case, the main source of IL-17 was the γδ T cells. These data reveal a dependence on the animal background and the infection dose, influencing the source of IL-17 that mediates *L. major* infection (42). In the context of low-dose infection, C57BL/6 mice vaccinated with a DNA vaccine based on thiol-specific-antioxidant (TSA) not only showed reduced lesion and parasitic load upon *L. major* challenge, but also exhibited an increase in γδ T cells *in situ* compared with each of the other groups, including the healed mice, when vaccinated mice were injected in the ear with *L. major*-soluble Ag (SLA, 12.5 μg) and live metacyclic promastigotes (10^6^) (119). Many researchers have focused on studying *L. major* infection due to its potential to reveal a balanced interaction between Th1/Th2/Th17 responses and regulatory responses involving IL-10, particularly with the strain WHO/MHOM/IL/80/Friedlin. In this context, it has been demonstrated that IL-17 mediates immunopathology in the absence of IL-10 during *L. major* infection (120). The blockade of IL-10 receptor (IL-10R) has been shown to increase the expression of IL-17A and IFN-γ. Moreover, IFN-γ inhibits neutrophil recruitment and IL-17 production after IL-10R blockade. Similarly, dual blockade of IL-10R and IFN-γ increases IL-17A expression, highlighting the delicate balance between these cytokines. The primary source of IL-10 was identified as CD4 T cells, with no significant effect observed in IL-10-producing γδ T cells. Furthermore, although not explicitly demonstrated, the authors mention that there was a slight increase in IL-17 production by CD8 and γδ T cells in IL-10-deficient mice compared to controls (120), suggesting that IL-10 may regulate the expansion of IL-17-producing γδ T cells in other *Leishmania* infection models.

Overall, these findings illustrate that γδ T cells play versatile and strain-dependent roles during *L. major* infection, acting as modulators of IL-4, IFN-γ, and IL-17 responses. Their influence on disease outcome appears tightly linked to cytokine context, genetic background, and infection dose, emphasizing their dual potential to drive either protection or pathology.

• **L. amazonensis**

The presence of γδ cells has been observed in the draining lymph nodes of mice for extended periods, exceeding 100 days post-infection (121, 122). Susceptible C57BL/10 mice exhibit a more intense presence of γδ T cells in the draining lymph nodes of the lesion compared to CBA/J and C3H.He mice, suggesting that γδ T cells may play a crucial role in susceptible mice (122). This prolonged and pronounced accumulation indicates that γδ T cells might participate in sustaining the chronic inflammatory process characteristic of *L. amazonensis* infection. Indeed, for the first time in experimental *L. amazonensis* infection, a correlation between IL-17 and γδ T cells was demonstrated (121). Susceptible Sv129 mice showed a higher frequency and number of IL-17-producing γδ T cells, more than BALB/c mice, in the draining lymph nodes of the lesion and these mice failed to induce an IFN-γ response by CD4 and CD8 T cells (121). This suggests that IL-17-producing γδ T cells may contribute to disease susceptibility by promoting a non-protective inflammatory environment.

In BALB/c mice infected with *L. amazonensis*, treatment with the antibody anti-γδ TCR UC7-13D5 two days before each dose of an intranasal vaccine blocked the protective effect of the vaccine, resulting in a higher parasitic load and more lesion size compared to untreated, vaccinated mice (123), demonstrated the importance of these cells in susceptible mice (**Table 2**). These results reinforce that γδ T cells play a positive role in vaccine-induced protection, likely by modulating early immune activation and effector mechanisms.

Indeed, γδ T cells, defined by the expression of Vγ4, are the primary sources of IL-17 in susceptible mice during *L. amazonensis* infection (12). Uninfected Sv129 mice exhibit two distinct populations of γδ T cells, namely CD3^High^ and CD3^Low^ (9, 11, 12). This same profile is also observed in PBMCs, defined by populations of Vδ1^+^ and Vδ2^+^ cells (13–16). Infection with *L. amazonensis* surprisingly induces an increase in IL-17-producing CD3^Low^ γδ T cells compared to uninfected mice (12). These findings reveal an important feature of γδ T cells in *L. amazonensis* infection, as previous studies have identified CD3^High^ γδ T cells as the largest producers of IL-17 (9, 11). Therefore, the shift toward IL-17 production by CD3^Low^ γδ T cells represents a functional reprogramming associated with disease susceptibility.

The type 1 interferon receptor was crucial in controlling the expansion of these cells (12), however, these effects appeared to be strain-dependent (124). This observation suggests that host genetic background influences the regulatory pathways that limit γδ T cell expansion and IL-17 production. Additionally, it was observed that LPG from *L. amazonensis* induces the expansion of these cells, suggesting it could act as a ligand for γδ T cells, similar to LPG from *L. mexicana* (32). Such interaction points to a possible direct recognition of parasite-derived molecules by γδ T cells, reinforcing their role as innate-like sensors of infection.

C57Bl/6 mice lacking γδ T cells or the IL-17 receptor develop smaller lesions, indicating that γδ T cells producing IL-17 contribute to the pathogenesis of cutaneous leishmaniasis. Furthermore, elastase-deficient mice exhibit the same lesion profile, indicating the existence of an axis between IL-17 produced by γδ T cells and neutrophil recruitment (12). In contrast, adoptive transfer of FACS-purified γδ T cells increases the presence of IFN-γ-producing γδ T cells, which helps control lesion progression (12). However, when IFN-γ-deficient γδ T cells are transferred, control over lesion development is lost. These findings highlight the dual role of γδ T cells in the disease, where IL-17A-producing γδ T cells drive pathogenesis, while IFN-γ-producing γδ T cells offer a potential therapeutic strategy in cutaneous leishmaniasis (12). Additionally, *L. braziliensis* infection seems to induce the expansion of IL-17-producing γδ T cells through an axis involving the skin microbiota (125); however, further studies are needed to elucidate this relationship. Collectively, these findings suggest that the γδ T cell–IL-17 axis represents a conserved mechanism of immune modulation in *Leishmania* infections, influenced by host genetics, parasite species, and microbial environment.

• L. mexicana

The presence of γδ T cells was also observed, with αβ T cells being prevalent in the granulomas of *L. mexicana*-infected C57BL/6 mice (46). However, despite not being demonstrated, the authors mention that they did not observe any differences in the frequency of γδ T cells in the ear between infected and non-infected CBA/J, C57BL/6, and BALB/c mice. Even though mice showed clear differences in lesion size and parasite load (126). This suggests that the local abundance of γδ T cells may not directly correlate with disease severity, implying that their functional activation, rather than mere frequency, could be the key determinant in infection outcome. It is important to emphasize that, although the same parasite inoculum was used, the infection route and the strain of *L. mexicana* parasite differed from the previous study (46, 126). These methodological variations may explain discrepancies between studies and highlight the influence of both parasite strain and infection site on γδ T cell responses.

Olguín et al. (32) demonstrated that after stimulation of γδ T cells with *L. mexicana* LPG, a pronounced co-localization of LPG with TLR2 was observed. Furthermore, phosphorylation of IκBα was detected following stimulation with both LPG and peptidoglycan (positive control), indicating that LPG is recognized by TLR2 in γδ T cells, which leads to their activation, as evidenced by phosphorylated IκBα (32). These observations reveal that *L. mexicana* LPG acts as a potent activator of γδ T cells through TLR2-mediated signaling, triggering downstream inflammatory pathways. After inoculating of *L. mexicana* promastigotes or LPG into the dermis of BALB/c mice ears, dermal γδ T cells were observed to be both TLR2^+^ LPG^+^ and TLR2^+^LPG^-^ in equal proportions when animals were injected with *L. mexicana* promastigotes. However, when injected with LPG, a higher frequency of TLR2^+^LPG^-^ γδ T cells was observed, highlighting the involvement of other receptors in LPG recognition. This finding suggests that multiple receptor pathways participate in γδ T cell activation during *L. mexicana* infection, reflecting the complexity of host–parasite molecular interactions.

γδ T cell numbers increased after LPG inoculation into the dermis, whereas inoculation with *L. mexicana* promastigotes or PBS did not induce significant changes (32). Peptidoglycan decreased the absolute number of γδ T cells, suggesting that competition may occur in terms of the activation level of γδ T cells, depending on which ligand activates TLR2. These data indicate that TLR2 engagement by different ligands can modulate the amplitude of γδ T cell responses, possibly shaping the balance between activation and tolerance in the skin microenvironment. Additionally, an increase in the frequency of γδ T cells expressing TNF was observed after stimulation with LPG or *L. mexicana*. Overall, these findings show that *L. mexicana* LPG is detected by TLR2 on γδ T cells, leading to an increase in γδ T cell numbers in the skin and a heightened expression of TNF (32). Taken together, these results demonstrate that LPG–TLR2 interactions represent a key pathway for γδ T cell activation during *L. mexicana* infection, linking parasite recognition to TNF-mediated inflammatory responses that may contribute to disease pathogenesis or control.

• L. guyanensis

Hartley et al. (44) discovered that *L. guyanensis*-RNA-virus 1 (LRV1) induces IL-17 secretion in both human and murine leishmaniasis, contributing to the severity of LRV1-mediated disease. CD4 and γδ T cells were identified as the primary sources of IL-17. This finding reinforces the idea that IL-17-producing lymphocytes, including γδ T cells, are key mediators of inflammation and tissue pathology in certain *Leishmania* infections.

It is important to highlight that LRV1-dependent IL-17A secretion is enhanced in the absence of IFN-γ, further demonstrating, as seen in *L. major* infection (120), that there is a regulatory balance between these cytokines, where one inhibits the other. Such reciprocal regulation between IL-17 and IFN-γ underscores the complexity of cytokine networks that shape disease outcomes, with the dominance of one pathway potentially dictating the severity of infection.

However, when specifically assessing the role of γδ T cells using γδ T cell-deficient mice, no differences were observed in lesion size or parasite load compared to WT mice infected with either LRV1^+^ or LRV1^-^ (**Table 2**). Notably, mice infected with LRV1^+^ exhibited greater lesion sizes and higher parasite loads compared to those infected with LRV1^-^. These data suggest that, in the absence of γδ T cells, compensatory effects from IL-17-producing CD4+ T cells may mediate disease pathogenesis.

• L. donovani

During *L. donovani* infection, there is a time point of IL-17 expression in both the spleen and liver (127, 128). Culture of splenocytes with *L. donovani* soluble antigen is capable of inducing high production of IL-17. Using IL-17-deficient mice, it was observed that there was lower parasite load in the spleen and liver compared to WT mice on days 15 and 30 post-infection. Additionally, γδ T cells were identified as the main sources of IL-17 (127, 128), with a secondary contribution from CD4 cells. These findings suggest that IL-17-producing γδ T cells play a significant role in modulating the early immune response during visceral leishmaniasis.

It seems that there is a dynamic in which IL-17 regulates IFN-γ production (44, 120), as IL-17-deficient mice showed increased IFN-γ levels by splenocytes at both infection time points, with CD4 cells being the primary source (127). In the absence of IL-17, a reduction in neutrophils (CD11b^+^Ly6C^med^Ly6G^+^) and inflammatory monocytes (CD11b^+^ Ly6C^hi^ Ly6G^−^) was also observed, highlighting an axis involving IL-17-producing γδ T cells that act on the recruitment of neutrophils and monocytes, contributing to susceptibility to *L. donovani* infection. Notably, aggregates of monocytes and neutrophils were found in WT mice at 15 dpi but not in IL-17-deficient mice (127). Together, these results indicate that IL-17 contributes to disease progression rather than protection, primarily by sustaining a pro-inflammatory environment that favors parasite persistence.

When using TCR δ−deficient mice infected with *L. donovani*, it was observed that they had reduced parasite loads in their livers 7 days later, compared to WT mice, suggesting a suppressive role for γδ T cells in *L. donovani* infection (128). However, the reduction in parasite load in TCR δ−deficient mice was not as significant as that seen in IL-17−deficient mice, suggesting that other important cellular sources of IL-17 exist in the liver during *L. donovani* infection (**Table 2**). This reinforces the notion that, although γδ T cells are a major source of IL-17, other immune populations may also contribute to the IL-17-driven pathology in visceral leishmaniasis.

To further demonstrate the suppressive role of IL-17-producing γδ T cells, mice reconstituted with 80% γδ−deficient Bone Marrow (BM) and 20% WT BM showed higher parasite loads compared to mice reconstituted with 80% γδ−deficient BM and 20% IL-17−deficient BM. These results support a suppressive role for IL-17 derived from γδ T cells in the liver during the early stages of *L. donovani* infection (128). Additionally, CD11b^+^Ly6C^hi^ monocytes recruited to the liver in response to *L. donovani* infection were found to have the highest expression of IL-17RA compared to other myeloid and non-myeloid cells, indicating that they are potentially important targets for this cytokine. These findings identify inflammatory monocytes as key mediators of IL-17–dependent immunosuppression in the liver.

Furthermore, CCR2-deficient mice, which have limited monocyte recruitment to infected and inflamed tissues, failed to improve parasite growth control after IL-17 blockade, but showed no defect in γδ T cell recruitment to the liver (128). These mice also had fewer inflammatory monocytes (CD11b^+^Ly6C^hi^) in the liver, suggesting that these monocytes are targets of the suppressive effect of IL-17. Furthermore, it has been speculated that parasite control was associated with the maintenance of mRNA expression of superoxide dismutase 3 in the absence of IL-17 in the liver (128). This suggests that IL-17 may interfere with oxidative stress responses critical for parasite clearance, further supporting its immunosuppressive role in visceral leishmaniasis.

It is interesting to note that during infection with the strain MHOM/SD/97/LEM3427, within the CD3^+^NK1.1^+^ population, no difference was observed in relation to cells expressing the γδ TCR (129). Hepatic resistance to *L. donovani* infection in mice is associated with the development of granulomas. Interestingly, B220^+^CD19^-^ cells were found within hepatic granulomas, and these cells expressed CD11c and/or γδ TCR (130), raising questions to be analyzed regarding the role of this cell subtype in the immune response. Altogether, these studies point to a complex and sometimes paradoxical role of γδ T cells during *L. donovani* infection, where IL-17 secretion may promote inflammation and cellular recruitment, yet ultimately contribute to immune suppression and parasite persistence.

## Discussion

The majority of γδ T cells recognize antigens independently of MHC, providing a potential advantage over αβ T cells (71). In addition, this advantage may also be reflected in the expansion of γδ T cells, possibly favored by the immunosuppressive environment resulting from CD4^+^ and CD8^+^ T-cell dysfunction during *Leishmania* infection. Indeed, *L. amazonensis* and *L. braziliensis* have been shown to induce the expression of exhaustion markers such as PD-1 on CD4^+^ and CD8^+^ T cells and PD-L1 on dendritic cells and neutrophils, impairing IFN-γ production through PD-1/PD-L1-dependent mechanisms (59–61). Although some PD-1^+^ γδ T-cell populations have been associated with reduced effector function (131–134), PD-1^+^ γδ T cells may exhibit a more pronounced cytotoxic and effector phenotype, indicating that PD-1 expression can reflect activation rather than exhaustion (135–138). These findings suggest that γδ T cells may benefit from, or adapt to, the immunosuppressive milieu established during *Leishmania* infection, however, further studies are required to confirm this relationship and to elucidate its actual impact during *Leishmania* spp. infection.

It is important to note that the heterogeneity of γδ T-cell subpopulations may differentially influence the outcome of various diseases, including cancer, malaria, and *Leishmania* infection (135, 139–141). Therefore, a subset-focused analysis is necessary to better understand their specific contributions. Although Vδ1 cells are predominantly found in the epidermis and Vδ2 cells in the dermis of lesions caused by *Leishmania* spp (37)., their individual roles during infection remain unclear. Single-cell RNA sequencing studies have shown that Vδ1 and Vδ2 γδ T cells share core cytotoxic signatures but also display distinct effector programs, with Vδ1 cells enriched for tissue-resident and stress-sensing functions, while Vδ2 cells exhibit higher proliferative and IFN-γ–mediated responses (142). Moreover, differential TCR Vγδ usage has been shown to distinguish protumor from antitumor γδ T-cell subsets in the intestine, highlighting how their functional polarization can either sustain or limit inflammation (139). Consistently, shifts in the frequency of Vδ1 and Vδ2 subsets have been associated with systemic inflammatory markers during HIV infection, reinforcing that γδ T-cell subset balance is dynamically regulated by the inflammatory environment (143). Together, these findings underscore the need to dissect the specific contribution of each γδ T-cell subset in *Leishmania* infection to fully understand their dual role in immunity and pathology.

In line with this, a recent study showed that this functional dichotomy significantly influences *L. amazonensis* infection in experimental models. Among γδ T cells, Vγ4^+^ cells were identified as the primary producers of IL-17, in contrast to Vγ1^+^ or Vγ1^−^Vγ4^−^ populations. Notably, IL-17 production was linked to disease pathogenesis, whereas IFN-γ production was associated with resistance to infection (12). Complementing this, PD-1 and TIM-3 have been shown to differentially regulate IL-17-producing γδ T cell subsets, with Vγ6^+^ cells expressing high PD-1 and Vγ4^+^ cells upregulating TIM-3 in response to inflammatory signals (144). Together, these findings highlight the importance of assessing checkpoint receptor expression and function in γδ T cells during leishmaniasis, which could provide insights into therapeutic strategies to enhance their protective immune responses.

The BTLA and PD-1 signaling pathways independently regulate the proliferation and cytotoxicity of human peripheral blood γδ T cells, with BTLA signaling being crucial for proliferation and PD-1 modulating cytotoxic functions, illustrating the intricate control of γδ T cell activity (145). Baseline plasma levels of soluble PD-1 (sPD-1) and BTN2A1 have been identified as predictive biomarkers for response to immunotherapy in melanoma, where lower sPD-1 and higher sBTN2A1 levels are associated with better outcomes, suggesting that these molecules play a role in modulating γδ T cell responses (146). γδ T cells are also able to suppress *Plasmodium falciparum* blood-stage infection through direct killing and phagocytosis, involving TCR-mediated degranulation and CD16-dependent phagocytosis of antibody-coated infected red blood cells (147). Notably, BTN2A1 expression is upregulated in infected red blood cells, implicating it in γδ T cell activation during infection (147). Building on these observations, strategies that engage γδ T cells via checkpoint modulation or antibody targeting have been developed in oncology, bispecific antibodies targeting Vγ2 TCR and PD-L1 enhance the anti-tumor response of Vγ2Vδ2 T cells, while the anti-BTN3A antibody ICT01 activates Vγ9Vδ2 T cell-mediated cytotoxicity (148, 149). Together, these studies illustrate how γδ T cell function can be precisely modulated through checkpoint and BTN pathways, highlighting the potential for similar approaches in infectious diseases. In the context of *Leishmania* infections, assessing the expression and regulation of BTLA, PD-1, and BTN molecules on γδ T cells could provide novel insights into their protective or pathogenic roles, offering opportunities to enhance host immunity against the parasite.

In leishmaniasis, T cell memory comprises both circulating and tissue-resident populations (TRM), and the several studies of David L. Sacks and Phillip Scott were pivotal in defining this landscape. Sacks and colleagues highlighted the role of circulating T cells and discussed how the maintenance of immunity may depend on the balance between long-lived central memory and the persistence of parasites that sustain effector responses (150). Moreover, they demonstrated that cutaneous infection with *L. major* can elicit protective memory capable of conferring heterologous immunity against visceral infection with *L. infantum*, indicating that memory responses generated in the skin, mediated by T cells, have the potential to protect distant organs such as the liver and spleen (151). In contrast, Phillip Scott’s group convincingly showed that CD4^+^ TRM established in the skin after *L. major* infection provide rapid protection at the challenge site by recruiting and activating inflammatory monocytes and limiting parasite burden (152, 153). Together, these findings suggest that optimal immunity to *Leishmania* relies on the coordinated action of both tissue-resident and circulating T cell compartments, a concept that may also extend to unconventional subsets such as γδ T cells, which bridge innate and adaptive memory-like responses. Supporting this idea, data showed that γδ T cells persist in the circulation of patients with leishmaniasis even after clinical cure (83). This persistence indicates that circulating γδ T cells may contribute to long-term immune surveillance and retain memory-like functions, complementing the protective roles of tissue-resident populations.

γδ T cells have emerged as key players in both adaptive and innate immune memory, emphasizes that γδ T cells can develop either pathogen-specific adaptive memory or pathogen-independent trained immunity, depending on the context and subset involved (Vγ9Vδ2 or Vδ1 in humans, Vγ6 or Vγ4 in mice) (154). In humans and non-human primates, adaptive-like memory has been most clearly characterized in the Vγ9Vδ2 subset. After *Mycobacterium tuberculosis* exposure or BCG vaccination, these cells undergo antigen-specific expansion and enhanced IFN-γ production upon re-stimulation, even in the absence of CD4^+^ T cell help (155, 156). Similarly, *Listeria monocytogenes* infection induces long-lived Vδ2 T cells with stronger recall effector functions (157), while *Plasmodium falciparum* infection or vaccination drives both Vδ1 and Vδ2 memory responses correlated with protection against malaria (158, 159). These findings reveal that γδ T cells can establish clonally focused, antigen-specific recall responses, similar to αβ T cell memory. In line with this concept, studies in *Leishmania* infection have reported localized expansions of specific γδ T cell clones within cutaneous lesions, suggesting that these cells may undergo antigen-driven selection in discrete microenvironments. Such spatially restricted oligoclonal patterns are consistent with the formation of adaptive-like memory populations capable of responding more efficiently upon re-exposure to parasite antigens (37, 70). Together, these observations support the view that γδ T cells in *Leishmania* infection not only act as early effectors but can also retain long-term functional imprinting, bridging innate and adaptive memory mechanisms within the tissue.

At the same time, γδ T cells can also acquire trained immunity, a form of innate memory characterized by epigenetic and metabolic reprogramming that enhances responsiveness to unrelated secondary challenges (154). For example, macaques vaccinated with attenuated *L. monocytogenes* showed Vδ2 T cell–dependent protection against subsequent *M. tuberculosis* infection, despite the antigenic disparity (160). Likewise, BCG vaccination in humans increased IFN-γ and TNF production by γδ T cells in response to both mycobacterial and non-mycobacterial stimuli, indicating a trained phenotype (161). Murine studies further support this duality: IL-17 producing Vγ4^+^Vδ4^+^ T cells persist after skin inflammation and mount robust secondary responses to TLR7/8 ligands (162), while intestinal Vγ4^+^Vδ1^+^ cells primed by *Listeria* exhibit cross-reactivity to unrelated bacteria (72, 163). In agreement with these findings, our data show that infection with *L. amazonensis* induces a high frequency of IL-17 producing Vγ4^+^ T cells, suggesting that similar mechanisms of functional reprogramming and persistence may occur during leishmaniasis (12, 121).

**Figure 4:**
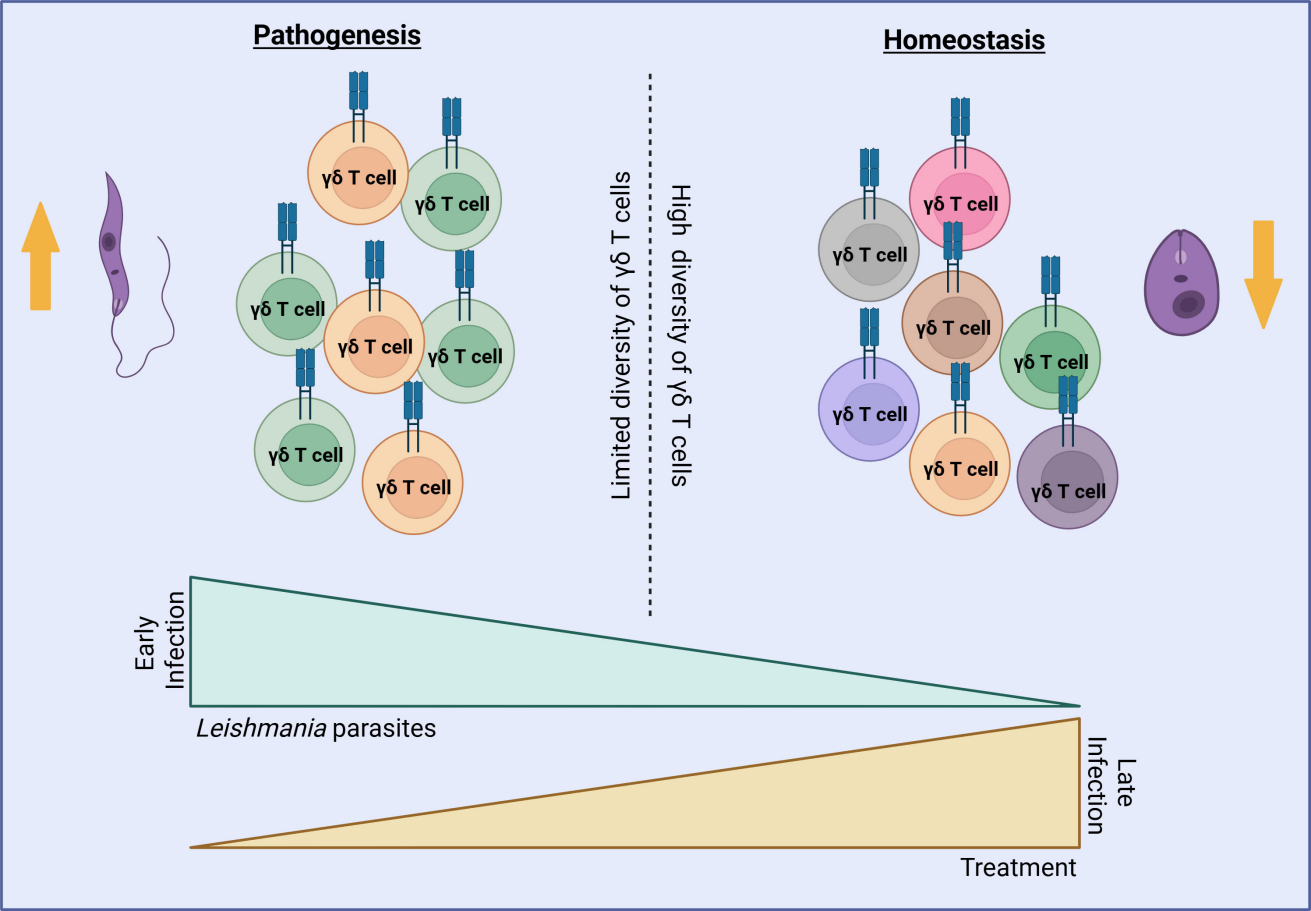
Proposed model for the limited diversity of T γδ cells in leishmaniasis. During the early phase of *Leishmania* infection, there is a greater availability of antigens that can be targeted for recognition by γδ T cells. Some antigens may be more dominant than others in the promastigote forms, leading to the selection of specific γδ T cell clones, thus creating a limited diversity that will act in the initial stages of infection related to the pathogenesis. However, after the onset and progression of treatment, the number of parasites decreases, along with the availability of antigens. As a result, there is a shift in the diversity of γδ T cell clones, leading to greater diversity and, consequently, the process of homeostasis.

In *Leishmania* infection, γδ T cell diversity appears to be restricted, likely due to the expansion of specific clones in response to antigens (37, 70). This raises questions about antigen recognition and potential therapeutic targets. Besides, there is an inverse correlation between the use of treatment for leishmaniasis and the expansion of γδ T cells (38, 55, 80, 81), which may suggest that during treatment, there is a reduction in parasite load and consequently a decrease in the availability of antigens which are abundant in amastigote forms (164–166), leading to a reduced expansion of γδ T cells (**Figure 4**). Additionally, further studies are needed to evaluate whether treatment for leishmaniasis impacts the limited diversity of γδ T cells.

It is important to emphasize that polymorphisms in the structure of glyconjugates can induce different immune response (165–172). Therefore, it is crucial to understand whether these polymorphisms trigger different responses from γδ T cells and select different clones. Moreover, there is a need for greater clarity regarding the effects of using only the promastigote or metacyclic forms, as many studies use a mixture of these forms, which exhibit different LPG structures (166). Additionally, there is a gap in studies that use metacyclic forms and saliva, as saliva also contains proteophosphoglycans that may activate γδ T cells (173). Stimulation of PBMCs with *L. donovani* heat killed does not induce γδ T cell proliferation, as observed with live *L. donovani* stimulation (41). One possible explanation for this difference could be a structural alteration in the glyconjugates of the parasites heat killed (174) or infected macrophages can activate γδ T cells using BTN2A1 and BTN3A1, which are relatives of the B7 family molecules CD80 and PD-L1 (23, 28–31). These molecules act as phosphoantigen sensors and are crucial for the expansion and reactivity of γδ T cells in humans, as observed in *P. falciparum*-infected patients (147). In contrast, experimental models such as mice possess three main genes, Btnl1, Btnl4, and Btnl6, that are closely related to Skint1, a molecule essential for the development of skin-resident γδ T cells (dendritic epidermal T cells (DETC)) (175). This allows for the evaluation of the impact of deficiencies in specific models, such as the Btnl1 and Btnl4 knockout models (175).

DETCs are key players in cutaneous immune surveillance (176, 177). In mice, DETCs form a specialized epidermal subset expressing a relatively invariant TCR γδ (typically Vγ5/Vδ1) that monitors keratinocyte health. These cells engage in constitutive interactions with keratinocytes via Skint1, a process termed “normality sensing.” This mechanism maintains DETCs in a poised state, preserving their transcriptional program, supporting epidermal barrier integrity, and enabling rapid innate-like responses to tissue stress or injury (177). In the context of cutaneous leishmaniasis, where *Leishmania* parasites invade the skin and induce local tissue stress and inflammation (178, 179), this surveillance mechanism may be particularly relevant. DETCs could detect early stress signals from infected keratinocytes or surrounding tissue, rapidly producing cytokines or growth factors that modulate local immune responses (180–182), recruit additional immune cells, or influence antigen presentation by APCs, resident T cells, neutrophils, and macrophages (34). By maintaining tissue homeostasis while remaining responsive to infection-induced perturbations, DETCs may play a critical role in shaping the early cutaneous immune environment during *Leishmania* infection. Despite this potential, their precise contribution to parasite control, lesion development, and the modulation of adaptive immunity in leishmaniasis remains largely unexplored, representing an important avenue for future investigation.

In conclusion, this review offers an in-depth examination of the pivotal role played by γδ T cells in leishmaniasis, thoroughly discussing the diverse factors that shape the immune response to *Leishmania* infection. It emphasizes the variability across *Leishmania* strains, γδ T cell subpopulations, and different mouse genetic backgrounds, factors that profoundly influence infection outcomes. By addressing these complexities, we aim to provide valuable insights that will contribute to the development of more targeted and effective therapeutic strategies against leishmaniasis.
